# Isorhamnetin from *Lycium ruthenicum* Murr. exhibits anti-inflammatory and anticancer activities *in vitro* via regulation of oxidative stress, mitochondrial function, and AKT signaling

**DOI:** 10.3389/fnut.2026.1750263

**Published:** 2026-07-23

**Authors:** Lei Jiang, Jideng Ma, Zhiyuan Li, Yuqi Li, Shihan Zhang, Huifeng Li

**Affiliations:** 1 Key Laboratory of Tibetan Medicine Research, Northwest Institute of Plateau Biology, Chinese Academy of Sciences, Xining, China; 2 Clinical Laboratory Department, Qinghai Provincial Peoples Hospital, Xining, China; 3 College of Agriculture and Animal Husbandry, Qinghai University, Xining, China

**Keywords:** anticancer activity, anti-inflammatory activity, cell apoptosis, isorhamnetin, LC-MS/MS, *Lycium ruthenicum* Murr.

## Abstract

Black goji berry (*Lycium ruthenicum* Murr.) has been recognized for its immune-regulating and anti-cancer potentials, but its key active components and underlying mechanisms remain unclear. Here, we applied LC–MS/MS to characterize the metabolite profile of *L. ruthenicum* and performed *in vitro* assays to evaluate its anti-inflammatory and anti-cancer activities. A total of 149 metabolites were identified, with organic acids, carbohydrates, and amino acid derivatives as the dominant components. Isorhamnetin was identified as a representative flavonoid. In LPS-stimulated Calu3 cells, isorhamnetin reduced the secretion and mRNA expression of pro-inflammatory cytokines (IL-6, TNF-alpha, IL-1beta). In cancer cells, isorhamnetin induces ROS accumulation, DNA damage, mitochondrial dysfunction, and caspase-3-dependent apoptosis, with modulation of the AKT signaling pathway. These findings suggest that isorhamnetin may contribute to the anti-inflammatory and anti-cancer effects of *L. ruthenicum* observed *in vitro*, providing preliminary experimental evidence for its potential as a functional food ingredient. However, this study is limited by the use of a single cell line (Calu3) and the absence of *in vivo* validation and pharmacokinetic evaluation; further studies are needed to confirm these observations.

## Graphical abstract

**Figure:**
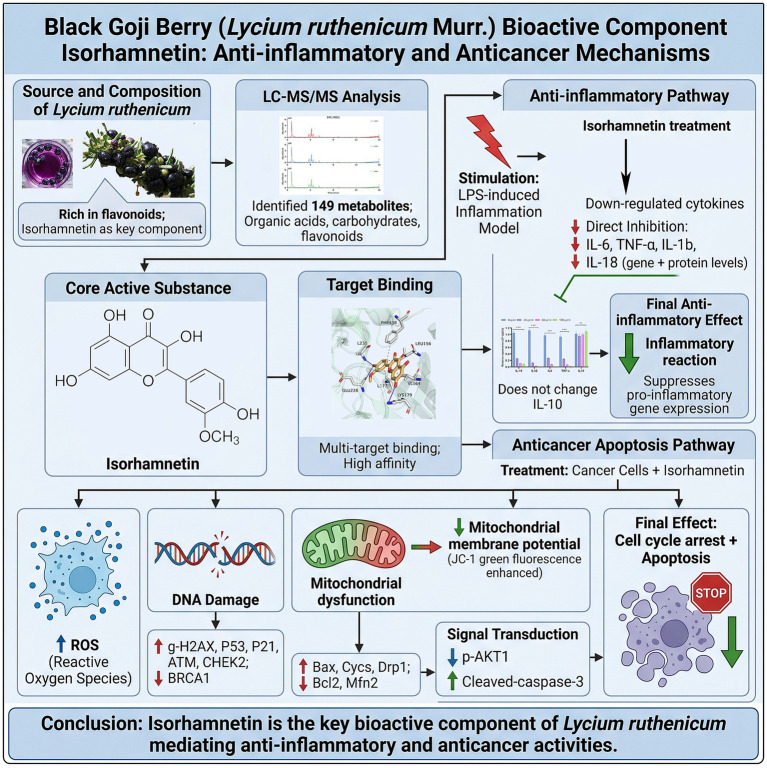

## 1 Introduction

In 2022, nearly 20 million new cancer cases and 9.7 million cancer deaths were recorded worldwide (1, 2), with chronic inflammation identified as a contributing factor in approximately 25% of all cancer cases. Despite significant advances in targeted therapy and immunotherapy, the global burden of cancer and inflammatory diseases continues to grow. Conventional treatments, while effective for many patients, are often accompanied by severe side effects, drug resistance, and prohibitive costs. These limitations have motivated an urgent search for safer, more accessible preventive and therapeutic strategies from natural sources (3, 4). Historically, natural products have provided some of the most impactful drugs in oncology—from paclitaxel to artemisinin—yet the vast majority of medicinal plants remain chemically and pharmacologically uncharacterized at the molecular level.

Among medicinal plants with documented health benefits, *Lycium ruthenicum* Murr., commonly known as black goji berry, has attracted considerable attention (5). Despite growing interest, the composition of *L. ruthenicum* at the level of individual compound identity and relative abundance has not been systematically characterized (5). Native to the Qinghai-Tibet Plateau and Central Asia, this fruit has been used in traditional Tibetan and Chinese medicine for centuries. Modern phytochemical investigations have identified over 100 bioactive constituents in *L. ruthenicum*, including anthocyanins, flavonoids, polysaccharides, phenolic acids, and alkaloids (6, 7). *In vitro* and *in vivo* studies have demonstrated that *L. ruthenicum* extracts exhibit antioxidant, anti-inflammatory, immunomodulatory, and antiproliferative activities (2, 8–11).

Which specific compound within this complex mixture drives these effects? Without an answer, reliable quality control standards and evidence-based dosage recommendations remain out of reach. Most prior studies of *L. ruthenicum* have used crude extracts, conflating dozens or hundreds of compounds and obscuring the identity of the true bioactive agent. Flavonoids—the most chemically diverse class with 34 identified compounds—are widely recognized for their anti-inflammatory and anticancer properties (12, 13), yet no study has pinpointed which flavonoid mediates the effects of this berry. A related gap is that the metabolite profile of *L. ruthenicum* has not been surveyed as a whole. Our metabolomic analysis reveals that the vast majority of *L. ruthenicum* constituents are common dietary metabolites—organic acids, sugars, and amino acid derivatives routinely found in conventional fruits and vegetables. This composition, dominated by ubiquitous food constituents, provides a chemical rationale for the safety of *L. ruthenicum* as a dietary berry.

Untargeted metabolomics and computational pharmacology now offer a way to bridge these gaps. LC-MS/MS can profile a plant metabolome comprehensively, while network pharmacology and molecular docking can prioritize compounds based on predicted target interactions (14). This integrated approach shifts the question from “Does the extract work?” to “Which compound works, through which target, and why?”.

In the present study, we employed this strategy to identify the bioactive flavonoid(s) of *L. ruthenicum*. We profiled the metabolome using LC-MS/MS, identifying 149 metabolites. Network pharmacology screened 54 orally bioavailable compounds against 630 protein targets, followed by molecular docking. This pipeline converged on isorhamnetin, a flavonoid that showed the strongest multi-target engagement with core cancer- and inflammation-related proteins (EGFR, AKT1, ESR1, CASP3) despite its modest relative abundance (0.01% of the total extract). We validated these predictions in Calu3 cells, confirming anti-inflammatory and pro-apoptotic effects through ROS accumulation, DNA damage, mitochondrial dysfunction, and AKT signaling modulation.

An important consideration when evaluating dietary plants is their safety profile. Unlike medicinal plants that may contain potent, sometimes toxic, secondary metabolites, *L. ruthenicum* has a long history of safe consumption as a conventional food ingredient (5). This distinction carries a practical implication: bioactive compounds in food plants typically exhibit moderate binding affinities and gentle regulatory effects on cellular pathways, which is appropriate for compounds intended for chronic low-dose dietary exposure rather than acute pharmacological intervention (15, 16). The concept of nutritional pharmacology recognizes that dietary bioactive often operate through network-wide modulation of multiple targets with moderate affinity, in contrast to the high-affinity single-target engagement typical of pharmaceutical drugs (17). The moderate IC50 of isorhamnetin (111.8 μg/mL) should therefore be interpreted in this context—as an expected profile for a food-derived compound whose safety allows long-term dietary use.

## 2 Materials and methods

### 2.1 Materials

HPLC-grade methanol, acetonitrile, and formic acid were purchased from Thermo Fisher Scientific (Waltham, MA, United States). Ultrapure water (18.2 MΩ·cm) was prepared using a Milli-Q system. Isorhamnetin (purity > 98%) was obtained from Sigma-Aldrich (St. Louis, MO, United States) and used for *in vitro* validation assays.

Authenticated *Lycium ruthenicum* Murr. Berries were artificially cultivated and collected in Qinghai Province, China, in 2024. Three independent biological replicates (from different batches of berries) were prepared for LC-MS/MS analysis. The samples were lyophilized and cryogenically pulverized with liquid nitrogen. For phytochemical characterization, 100 mg of pulverized berries was extracted with 900 μL ice-cold methanol: water (7:3, v/v) containing 4 μg/mL deuterated internal standards. The mixture was homogenized by vortex, pre-cooled at −40 degree C for 2 min, mechanically disrupted at 60 Hz for 2 min, sonicated in an ice-water bath for 60 min, incubated at −40 degree C for 30 min, and centrifuged at 12,000 rpm, 4 degree C for 10 min. The supernatant was diluted 10-fold and filtered through 0.22-μm nylon membranes before LC-MS/MS analysis. Metabolite identification followed a stringent two-step filtering protocol using an in-house MS/MS database of authenticated standards: Jiang et al. (7) the precursor m/z of each compound in full-scan MS must exactly match the corresponding standard (mass error < 1 ppm); Lu et al. (18) the diagnostic fragment ions in the MS/MS spectrum must correspond one-to-one with the major fragment peaks of the authentic standard. Only compounds satisfying both criteria were accepted as valid identifications. Over 4,000 initial MS features were detected; after applying this rigorous filtering, the six most abundant compounds and rutin were confirmed by standard matching, and all compounds that failed to meet both criteria were excluded. The complete list of validated metabolites is provided in Supplementary Table S1.

Metabolite identification followed a stringent two-step filtering protocol using an in-house MS/MS database of authenticated standards. First, the precursor m/z of each compound must exactly match the standard (mass error < 1 ppm). Second, the diagnostic MS/MS fragment ions must correspond one-to-one with major fragment peaks of the authentic standard (Supplementary Figure S5). Six high-abundance metabolites and rutin were confirmed by direct standard matching (Level 1 identification according to the Metabolomics Standards Initiative guidelines). All other reported metabolites were assigned at Level 2 (putatively identified based on MS/MS spectral matching with public databases) (Supplementary Table S1).

Lipopolysaccharide (LPS, Sigma-Aldrich), ROS fluorescence probe DCFH-DA, *γ*-H2AX immunofluorescence kit, JC-1 mitochondrial membrane potential assay kit (all from Beijing Solarbio), MTT reagent, and primary antibodies targeting p-p65, p-AKT1, caspase-3, cleaved-caspase3 and *β*-actin (Cell Signaling Technology) were used. Calu3 cells and human bone marrow mesenchymal stem cells (hMSCs) applied in this study were purchased from Procell Life Science & Technology Co., Ltd., Wuhan, China. Shanghai Fuheng Biotechnology Co., Ltd. only performed STR identification testing for Calu3 cells and was not the cell supplier. The STR authentication report of Calu3 cells and CD90 immunofluorescence identification report of hMSCs are provided in Supplementary Files S1, S2, respectively. All cells were cultured in RPMI 1640 medium supplemented with 10% fetal bovine serum (FBS) and 1% penicillin–streptomycin at 37 °C with 5% CO₂.

Instrumentation included an ultrasonic homogenizer (F-060SD, FEYO), vortex mixer (TYXH-I, Hanuo), refrigerated centrifuge (TGL-16MS, Luxiangyi), UPLC-Q-Exactive HF system (Waters ACQUITY UPLC I-Class with HSS T3 column (100 mm × 2.1 mm, 1.8 μm) coupled to a Thermo Q-Exactive HF Orbitrap mass spectrometer with HESI ion source), fluorescence microscope (Olympus IX73), microplate reader (Bio-Rad), and Western blot system (Bio-Rad).

### 2.2 Liquid chromatography-mass spectrometry conditions

Chromatographic separation was performed on a Waters ACQUITY UPLC I-Class HF system with an HSS T3 column (100 mm × 2.1 mm, 1.8 μm) maintained at 45 °*C. mobile* phase was (A) water with 0.1% formic acid and (B) acetonitrile; flow rate 0.35 mL/min. Gradient elution: 95% A/5% B (0–2 min); linear transition to 70% A/30% B (4 min); 50% A/50% B (8 min); 20% A/80% B (10 min); 0% A/100% B (14–15 min); re-equilibration to 95% A/5% B (15.1–16 min). Injection volume was 5 μL.

Mass spectrometry was conducted on a Thermo Q-Exactive™ Orbitrap spectrometer with a HESI source (positive/negative modes). Full scan MS data (m/z 100–1,200) were acquired at 70,000 resolution, followed by DDA (top 8 precursor ions) with stepped NCEs (10, 20, 40 eV) at 17,500 MS/MS resolution. Ion source parameters: spray voltage (+3,800 V positive/−3,000 V negative); capillary temperature 320 °C; auxiliary gas heater 350 °C; sheath gas 35 arb; auxiliary gas 8 arb; S-lens RF level 50.

### 2.3 Target prediction

*Lycium ruthenicum* bioactivity was computationally assessed via a multi-step workflow. Identified phytochemicals were filtered for oral safety using RDKit, retaining compounds meeting Lipinski’s Rule of Five (MW < = 500 Da, LogP <= 5, HBA < = 10, HBD < = 5) with no structural alerts. Target prediction used two complementary approaches: experimentally validated targets from BATMAN-TCM 2.0^1^ via PubChem IDs; novel targets from NetInfer^2^ with confidence score > = 1. Network pharmacology was used as a preliminary screening tool for candidate prioritization, not as a definitive method for confirming target-drug efficacy. Isorhamnetin was the only compound with notable multi-target engagement among the 54 orally bioavailable candidates; the other compounds yielded no meaningful predictions. If stricter filtering criteria were applied at this stage, no candidate would remain for experimental validation. The current strategy provides sufficient rationale for the subsequent *in vitro* experiments. Targets were deduplicated, and compound-target networks were constructed using R packages igraph and ggnetwork.

### 2.4 Analysis of the target interaction network

PPI analysis was performed using STRING 12.0. The human PPI network was downloaded, and 630 *Lycium ruthenicum*-associated targets were mapped onto it, retaining interactions with a confidence score ≥ 700. The PPI network was visualized via igraph and ggnetwork. Topology metrics (degree, betweenness, and closeness centrality) were calculated, and core targets were the top 10 nodes by degree centrality.

### 2.5 Functional enrichment analysis of targets

Functional enrichment was performed using the R package clusterProfiler (v4.0) with GO (biological processes), KEGG (pathways, v102.1), and DISEASES (disease-gene associations, integrated score ≥ 2.0). Input was 559 PPI-mapped targets. Significance was determined by hypergeometric testing (FDR < 0.05). Visualizations included top 10 GO terms (bar plots), top 20 KEGG pathways (bubble plots), and top 20 diseases (dot plots).

### 2.6 Molecular docking of core targets related to the components of black wolfberry

A tripartite network (10 core targets, top 20 KEGG pathways, diseases) was constructed using igraph. Compounds targeting > = 2 core targets were visualized via bar plots. Molecular docking was performed with AutoDock Vina (v1.2.0) using isorhamnetin and core targets (EGFR: AlphaFold AF-P00533, AKT1: AF-P31749, TNF: AF-P01375, ESR1: AF-P03372, CASP3: AF-P42574). AlphaFold-predicted structures were used where experimental crystal structures were unavailable; while these models generally provide reliable folding predictions, they may not fully represent biologically active conformational states or ligand-induced conformational changes. Binding pockets were predicted with p2rank, docking grids (20x20x20 Angstrom, exhaustiveness = 8) centered on predicted pocket centroids. Clustering RMSD values between docking poses were calculated (Supplementary Table S2). Top-ranked poses were selected based on the lowest binding energy (kcal/mol). All docking poses were visualized and analyzed in PyMOL.

### 2.7 Cell experiment methods

#### 2.7.1 Cell culture and treatment

**Figure 1:**
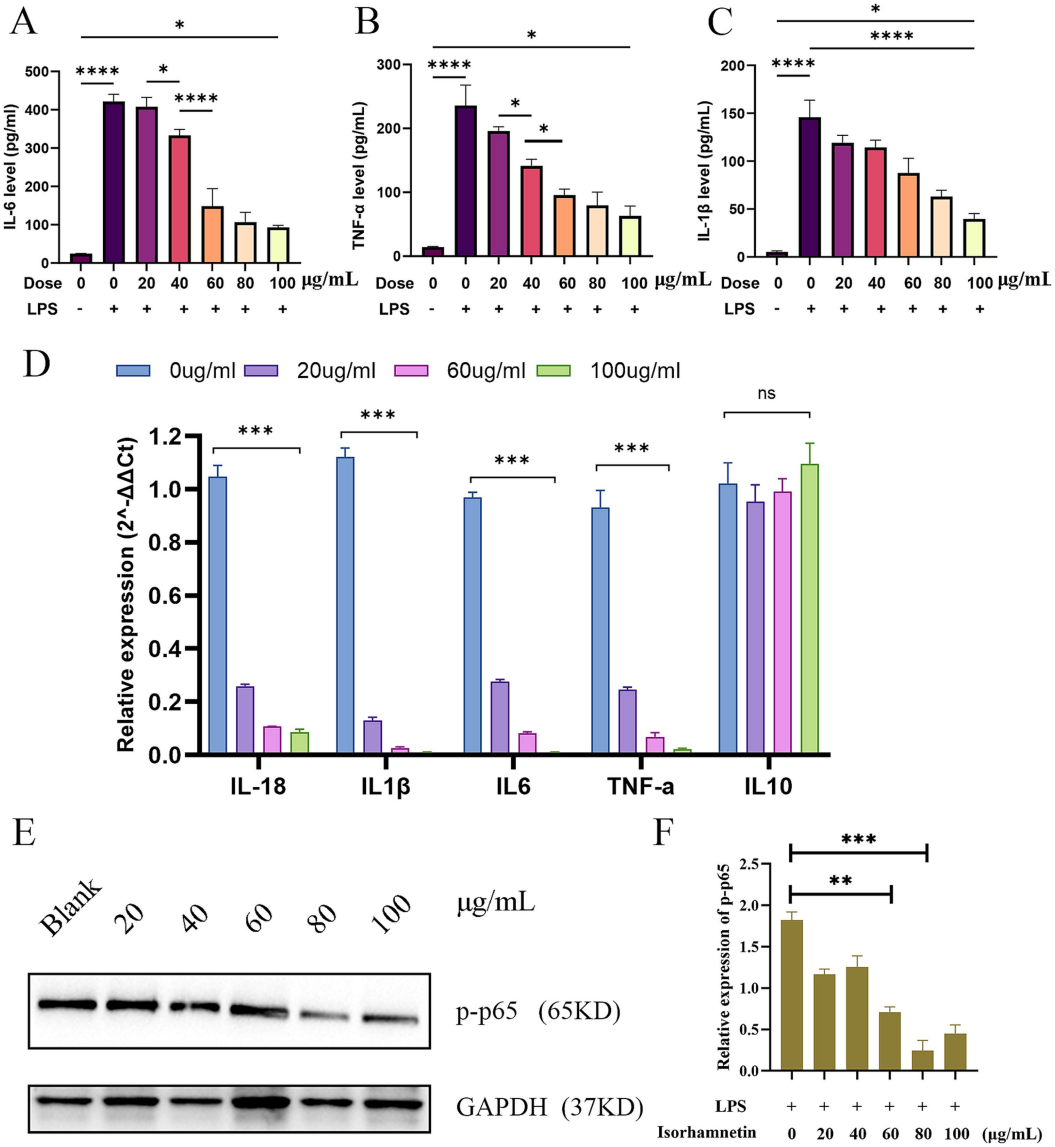
*In vitro* anti-inflammatory effects of isorhamnetin in lipopolysaccharide (LPS)-stimulated Calu3 human lung epithelial cells. **(A–C)** Effects of isorhamnetin (20, 40, 60, 80, 100 μg/mL, 24 h) on secretion of pro-inflammatory cytokines **(A)** IL-6, **(B)** TNF-*α*, and **(C)** IL-1*β* in LPS-stimulated (1 μg/mL, 24 h) Calu3 cells, measured by ELISA. **(D)** Relative mRNA expression of pro-inflammatory genes (IL-18, IL-1β, IL-6, TNF-α) and the anti-inflammatory gene IL-10 determined by RT-qPCR (*n* = 3). GAPDH was used as the internal reference gene. **(E,F)** Western blot analysis of NF-κB signaling: **(E)** representative bands of phosphorylated p65 (p-p65) and total p65; **(F)** densitometric quantification of p-p65 normalized to β-actin. Isorhamnetin significantly and dose-dependently suppressed p-p65 levels. All data are presented as mean ± SD (*n* = 3 independent experiments). *p < 0.05, **p < 0.01, ***p < 0.001, ****p < 0.0001 compared with the LPS model group by one-way ANOVA with Tukey’s post-hoc test.

**Figure 2:**
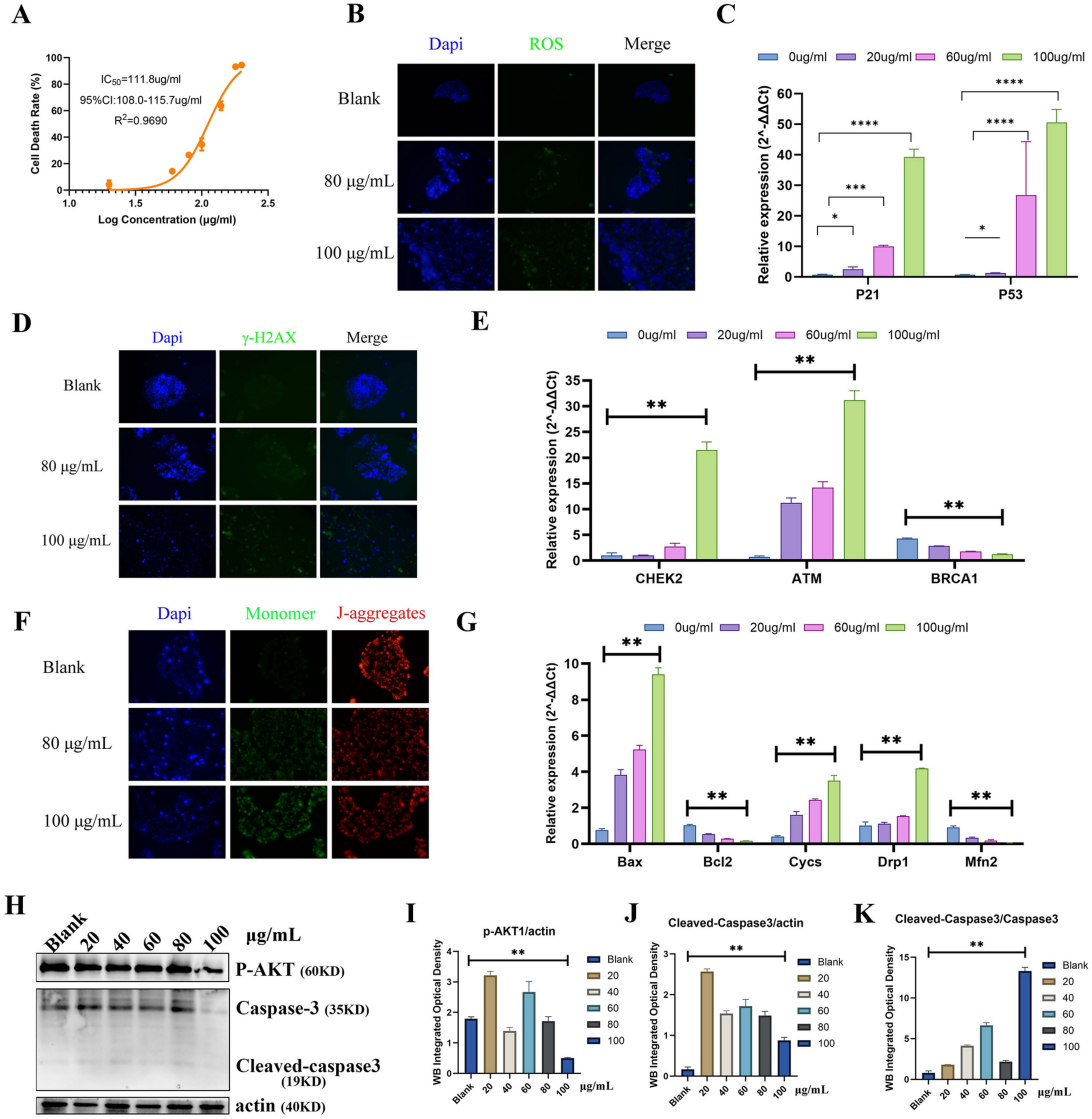
*In vitro* anticancer and pro-apoptotic effects of isorhamnetin in Calu3 human lung adenocarcinoma cells. Calu3 cells were treated with isorhamnetin (0, 20, 40, 60, 80, 100, 140, 180, 200 μg/mL) for 24 h. **(A)** Cell viability measured by the MTT assay. **(B)** Intracellular reactive oxygen species (ROS) levels detected by DCFH-DA fluorescence staining; green fluorescence intensity indicates ROS accumulation. **(C)** Relative mRNA expression of cell cycle regulatory genes P21 and P53 by RT-qPCR. **(D)** DNA damage assessed by *γ*-H2AX immunofluorescence staining; green foci indicate DNA double-strand breaks, counterstained with DAPI (blue). **(E)** mRNA expression of DNA damage response genes CHEK2, ATM, and BRCA1 by RT-qPCR. **(F)** Mitochondrial membrane potential (ΔΨm) measured by JC-1 staining; a decreased red/green fluorescence ratio indicates mitochondrial depolarization. **(G)** mRNA expression of mitochondrial apoptosis-related genes (pro-apoptotic: Bax, Cycs, Drp1; anti-apoptotic: Bcl2, Mfn2) by RT-qPCR. **(H–K)** Western blot analysis of AKT signaling and apoptosis execution: **(H)** representative bands; **(I)** densitometric quantification of phosphorylated AKT1 (p-AKT1) normalized to β-actin; **(J)** cleaved caspase-3 normalized to total caspase-3; **(K)** cleaved caspase-3 normalized to β-actin. All data are presented as mean ± SD (*n* = 3 independent experiments). *p < 0.05, **p < 0.01, ***p < 0.001, ****p < 0.0001 compared with the untreated control group by one-way ANOVA with Tukey’s post-hoc test.

Calu3 cells were seeded in 96-well or 6-well plates and cultured to 70–80% confluence. Based on the network pharmacology prediction that identified isorhamnetin as the top bioactive compound engaging multiple core targets (EGFR, AKT1, ESR1, CASP3), we used isorhamnetin (purity >98%, Sigma-Aldrich) for subsequent *in vitro* validation. For anti-inflammatory experiments (Figure 1), cells were pre-treated with LPS (1 μg/mL) for 24 h to establish an inflammatory model, then treated with different concentrations of isorhamnetin (20, 40, 60, 80, 100 μg/mL) for another 24 h. For cell viability, oxidative stress, DNA damage, mitochondrial dysfunction, and apoptosis experiments (Figure 2), cells were directly treated with isorhamnetin (0, 20, 40, 60, 80, 100 μg/mL) for 24 h, with blank medium as control.

#### 2.7.2 Cytokine detection

The concentrations of IL-6, TNF-*α*, and IL-1beta in cell supernatants were detected using ELISA kits according to the manufacturer’s instructions, with three technical replicates per group. Total RNA was extracted from cells using Trizol reagent, reverse-transcribed into cDNA. RT-qPCR was performed using SYBR Green PCR Master Mix on a qPCR instrument. GAPDH was used as an internal reference, and relative gene expression was calculated using the 2^(-DeltaDeltaCt) method. Primer sequences were designed for target genes (IL-18, IL1beta, IL6, TNF-alpha, IL10, P21, P53, CHEK2, ATM, BRCA1, Bax, Cycs, Drp1, Bcl2, Mfn2). All primer sequences are listed in Supplementary Table S3.

#### 2.7.3 Western blot detection

Total protein was extracted from cells using RIPA lysis buffer (with protease and phosphatase inhibitors), and quantified by the BCA method. Equal amounts of protein were separated by SDS-PAGE, transferred to PVDF membranes, blocked with 5% skimmed milk, incubated with primary antibodies at 4 °C overnight, then with secondary antibodies at room temperature for 1 h. Bands were visualized using an ECL chemiluminescence reagent, and densitometric analysis was performed using ImageJ software, with *β*-actin as an internal reference.

#### 2.7.4 MTT assay

Cells were seeded in 96-well plates, treated as described, then 20 μL MTT reagent (5 mg/mL) was added to each well and incubated for 4 h. The supernatant was discarded, 150 μL DMSO was added to dissolve formazan crystals, and absorbance was measured at 490 nm using a microplate reader to calculate cell mortality.

#### 2.7.5 ROS detection

Cells were loaded with 10 μM DCFH-DA ROS fluorescence probe (Beijing Solarbio Science & Technology Co., Ltd., Beijing, China) at 37 °C for 30 min, washed with PBS, and observed under a fluorescence microscope. Fluorescence intensity was quantified using ImageJ software.

#### 2.7.6 *γ*-H2AX immunofluorescence

Cells were fixed with 4% paraformaldehyde, permeabilized with 0.1% Triton X-100, blocked, and incubated sequentially with a γ-H2AX primary antibody and a fluorescent secondary antibody using a γ-H2AX immunofluorescence kit (Beijing Solarbio Science & Technology Co., Ltd., Beijing, China). Nuclei were counterstained with DAPI, and images were captured under a fluorescence microscope.

#### 2.7.7 Mitochondrial membrane potential detection

Cells were stained with JC-1 mitochondrial membrane potential assay kit (Beijing Solarbio Science & Technology Co., Ltd., Beijing, China) at 37 °C for 20 min, washed with PBS, and observed under a fluorescence microscope. The red/green fluorescence ratio was calculated to evaluate mitochondrial depolarization.

### 2.8 Statistics

All data were analyzed with *p* < 0.05 as the significance threshold. LC-MS data (triplicate technical replicates from each of three biological replicates) were expressed as mean±SD. Multiple groups were compared via one-way ANOVA (Tukey’s post-hoc test); two groups via Student’s *t*-test (normal distribution) or the Mann–Whitney *U* test (non-normal). Correlations were used Pearson’s (parametric) or Spearman’s (non-parametric) coefficients. Enrichment analyses included Benjamini-Hochberg correction. Network metrics were descriptively analyzed. Docking binding energies were evaluated via descriptive statistics and paired t-tests where applicable. Analyses were performed using GraphPad Prism v9.0 or R v4.2.0, with relevant R packages detailed previously. Effect sizes and 95% confidence intervals are reported in the figure legends for key comparisons; error bars represent SD or SEM as indicated in each figure legend.

## 3 Results

### 3.1 Analysis of compound composition

**Figure 3:**
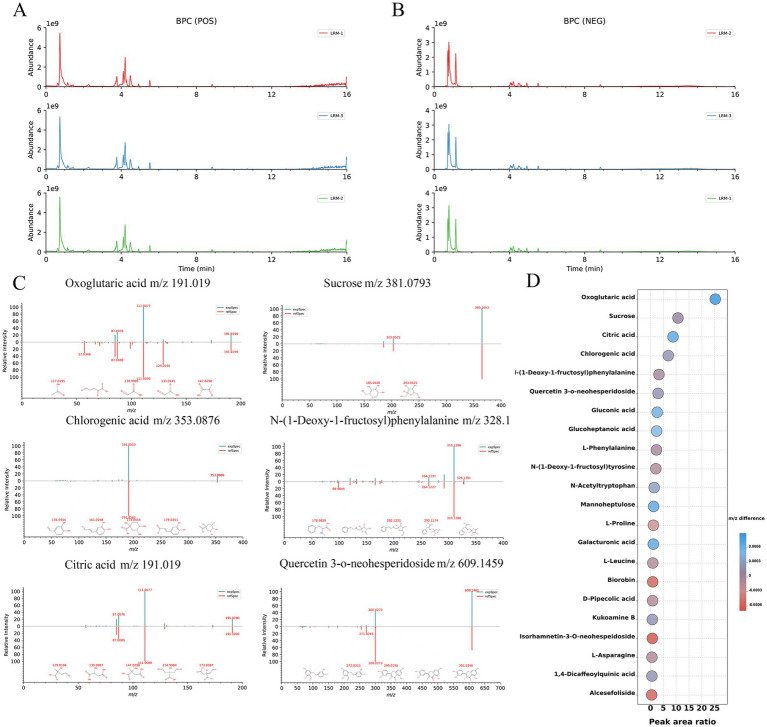
LC-MS/MS metabolite profiling of *Lycium ruthenicum* Murr. **(A,B)** Base peak chromatograms (BPCs) of *Lycium ruthenicum* Murr. berry extract showing complementary ionization profiles. **(A)** Negative mode (NEG), with intense signals in the early to mid-retention phase (0.6–2.2 min). **(B)** Positive mode (POS), with peak predominance in the mid-late retention window (3.5–5.5 min). **(C)** Identification of six representative high-abundance metabolites by matching with authenticated chemical standards. Identification criteria: precursor m/z mass error <1 ppm and diagnostic fragment ions matching standard spectra. **(D)** Abundance distribution of the 149 identified metabolites across chemical classes. Organic acids and carbohydrates dominate (>60% of aggregate peak area); the top 20 metabolites account for >77% of total signal intensity; specialized secondary metabolites are present at trace levels. All 149 metabolites were identified from three independent biological replicates of *L. ruthenicum* berries, validated by exact mass measurement, retention time consistency, and MS/MS fragmentation matching. The combined peak area of identified metabolites represents 87.66% of the total detected signal. Identification confidence follows Metabolomics Standards Initiative guidelines: level 1 (authenticated standards) for 7 compounds, level 2 (putative identification) for the remainder.

Liquid chromatography–tandem mass spectrometry (LC-MS/MS) identified 149 distinct metabolites following a stringent two-step filtering protocol. In brief, over 4,000 initial MS features were detected; each was filtered by precursor m/z matching with authenticated standards (mass error < 1 ppm) and one-to-one correspondence of diagnostic MS/MS fragment ions with standard spectra. Six high-abundance compounds and rutin were confirmed at the highest confidence level (Level 1) using authenticated chemical standards (Figure 3C). The remaining metabolites were assigned at Level 2 (putatively identified based on MS/MS spectral matching with public databases), in accordance with the Metabolomics Standards Initiative guidelines. The combined peak area of these metabolites accounted for 87.66% of the total detected signal. Base peak chromatograms (Figures 3A,B) showed complementary ionization profiles: negative mode (NEG) covered broad metabolites with intense signals at 0.6–2.2 min, while positive mode (POS) exhibited predominant peaks at 3.5–5.5 min. The metabolic profile was heterogeneous: organic acids and carbohydrates dominated (>60% aggregate peak area), with the top 20 metabolites contributing >77% of total signal intensity (Figure 3D). Isorhamnetin was detected at a relative abundance of 0.01% of the total extract (Supplementary Information Table S1).

### 3.2 Metabolite classification analysis reveals functional hierarchy

**Figure 4:**
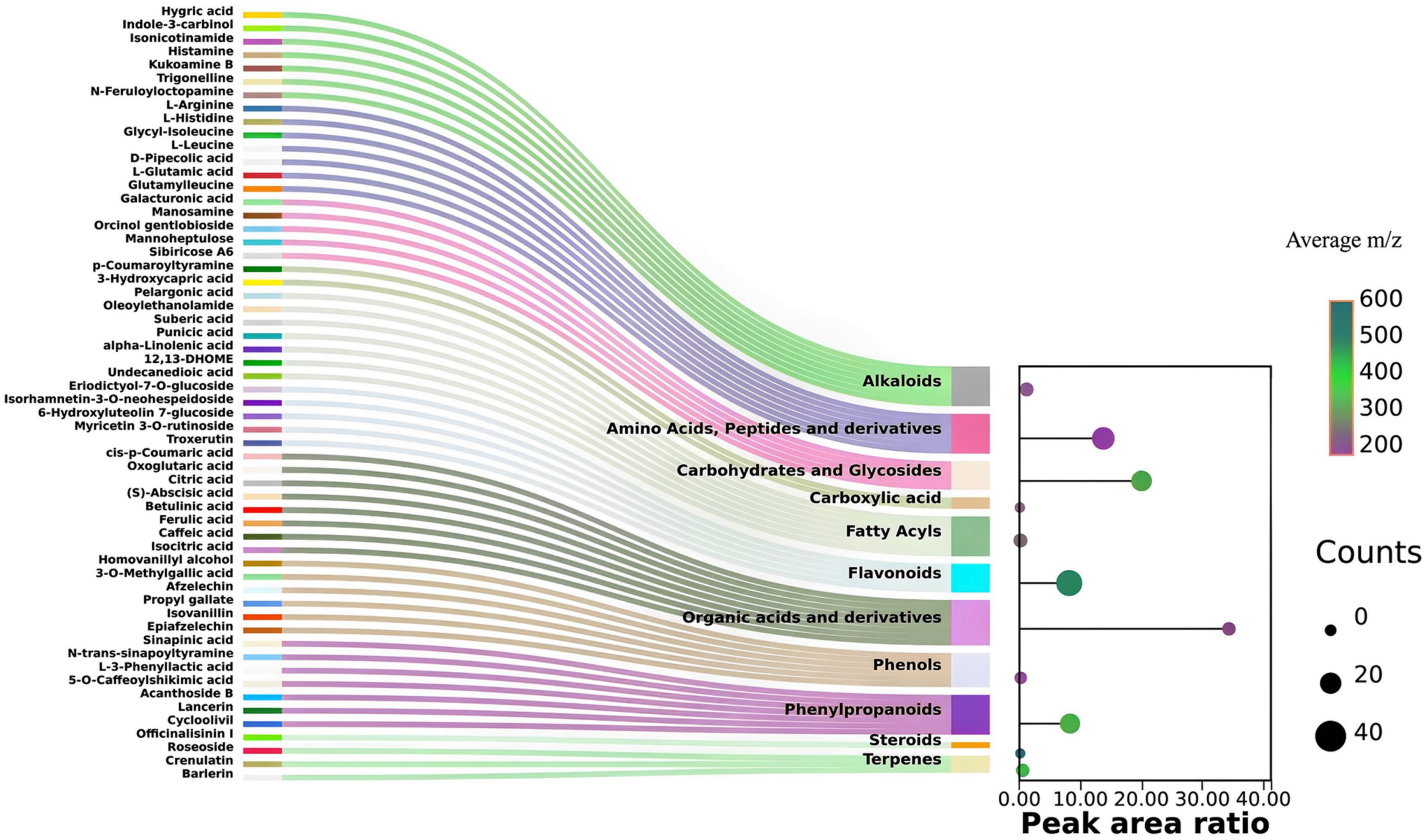
Metabolite classification and compositional characteristics of *Lycium ruthenicum* Murr. Left panel: Sankey diagram illustrating the mapping of 149 individual identified metabolites (left) to their respective chemical classes (right). Ribbon width is proportional to the peak area contribution of each metabolite to its class, representing relative abundance. Right panel: Bubble plot showing chemical class distribution. X-axis: total peak area per chemical class (overall abundance). Y-axis: average m/z per class (molecular weight range). Bubble size: number of metabolites per class (chemical diversity). Color gradient: average m/z range. Organic acids and derivatives dominate by total peak area despite comprising only 8 compounds; flavonoids show the highest chemical diversity (34 compounds) with moderate total abundance (8.17% of total peak area) (*n* = 3 biological replicates).

Metabolite classification (Figure 4) showed significant compositional heterogeneity. Organic acids and derivatives dominated (34.36% of the total peak area) with only 8 compounds, followed by carbohydrates and glycosides (20.03%, *n* = 23) and amino acids/peptides/derivatives (13.76%, *n* = 27). Flavonoids were the most diverse class (*n* = 34) but contributed moderately (8.17% of abundance). Despite this moderate contribution, flavonoids were prioritized for further investigation because: (i) they represent the most chemically diverse secondary metabolite class, (ii) numerous studies have established structure–activity relationships for flavonoid-mediated anti-inflammatory and anticancer effects, and (iii) network pharmacology analysis provided testable target predictions. Steroids had the highest median molecular weight (903.49 Da), followed by terpenes (353.92 Da) and phenylpropanoids (340.11 Da), whereas phenol derivatives were the lightest (205.07 Da). Among secondary metabolites, phenylpropanoids contributed to diversity (*n* = 22, 8.31% abundance), whereas fatty acyls (0.18%, *n* = 9), alkaloids (1.19%, *n* = 9), and carboxylic acids (0.07%, *n* = 2) were minor components.

### 3.3 Target prediction

**Figure 5:**
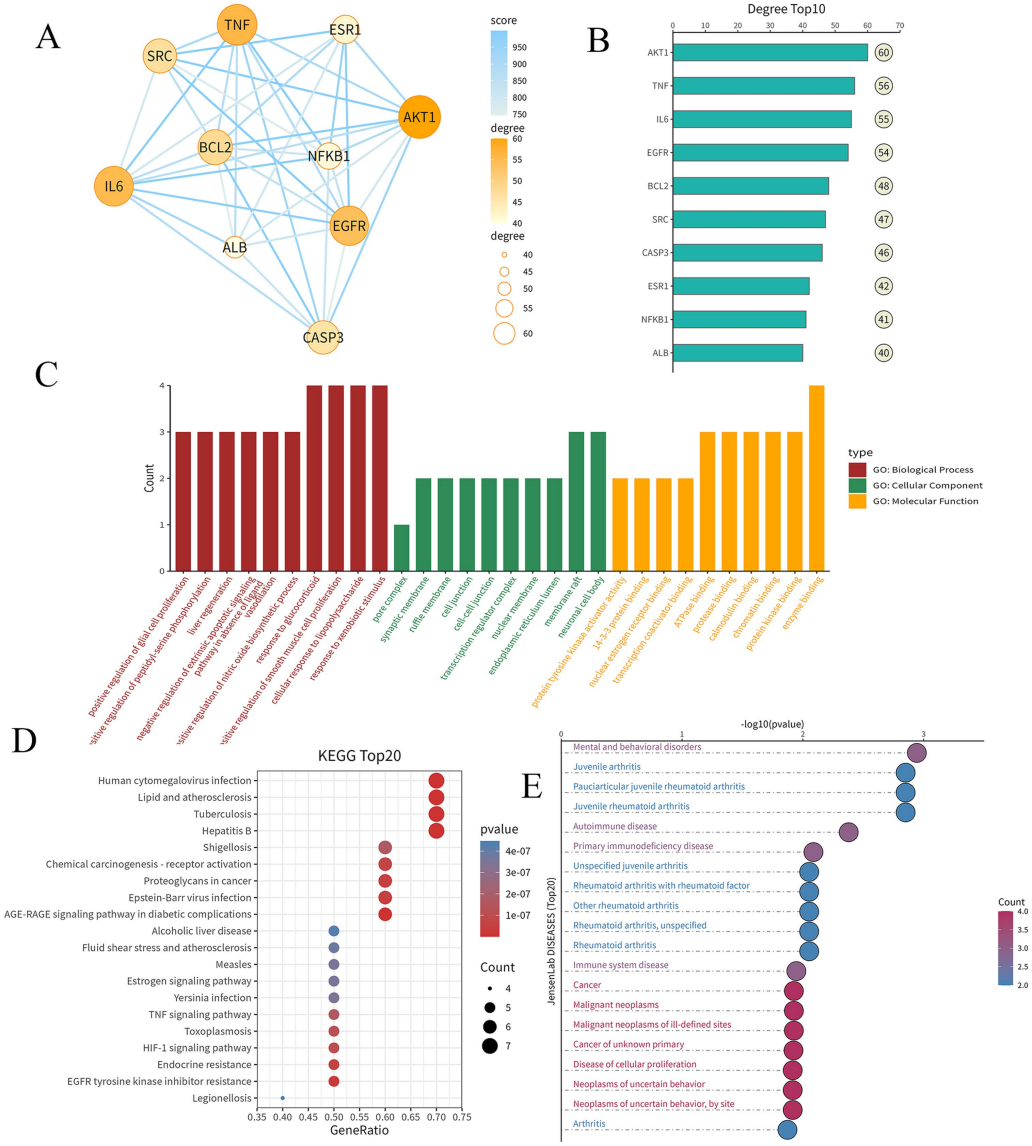
Core target network analysis of *Lycium ruthenicum* Murr. **(A)** Protein–protein interaction (PPI) subnetwork of the 10 core hub targets. **(B)** Top 10 core hub targets ranked by degree centrality; AKT1, TNF, and IL6 show the highest connectivity. **(C)** Gene Ontology (GO) biological process enrichment analysis of core targets (FDR < 0.05). **(D)** KEGG pathway enrichment analysis of core targets (FDR < 0.05), showing the top 20 enriched pathways. **(E)** Disease association analysis linking core targets to inflammation- and cancer-related disease categories (the compound-target interaction network and full PPI network analyses are presented in Supplementary Figures S1, S2, respectively).

RDKit-based screening identified 54 orally bioavailable compounds from *Lycium ruthenicum*, all complying with Lipinski’s rules and structural safety thresholds. Integrative target mining yielded 630 unique protein targets: 236 experimentally supported by BATMAN-TCM and 456 high-confidence predictions from NetInf (score > = 1). A compound-target interaction network was constructed (Supplementary Figure S1; Supplementary materials). STRING database mapping showed that 559 of the 630 targets (88.7%) formed high-confidence interactions (score ≥ 700). Degree centrality analysis identified 10 core targets. AKT1 had the highest degree (60), followed by TNF (56) and IL-6 (55) (Figures 5A,B; Supplementary Figure S2; Table 1). AKT1 and TNF also exhibited high betweenness centrality, whereas ALB showed high closeness centrality. Enrichment analysis revealed core functions, including glucocorticoid response, smooth muscle proliferation regulation, and lipopolysaccharide response (Figure 5C). KEGG pathways were enriched in viral infections, metabolic-inflammation crosstalk, and EGFR tyrosine kinase inhibitor resistance (Figure 5D). Disease associations prioritized autoimmune disorders (especially rheumatoid arthritis), psychiatric conditions, and primary immunodeficiencies (Figure 5E).

**Table 1:** Core target network properties.

Targets	Degree	Betweenness	Closeness
AKT1	60	7783.774	0.000706
TNF	56	6789.338	0.000708
IL6	55	8633.387	0.000711
EGFR	54	6683.439	0.000696
BCL2	48	4887.137	0.000671
SRC	47	7121.88	0.000683
CASP3	46	5409.022	0.000685
ESR1	42	6960.607	0.000681
NFKB1	41	2505.142	0.000659
ALB	40	11471.58	0.00071

### 3.4 Molecular docking of core target molecules related to black goji berry components

**Figure 6:**
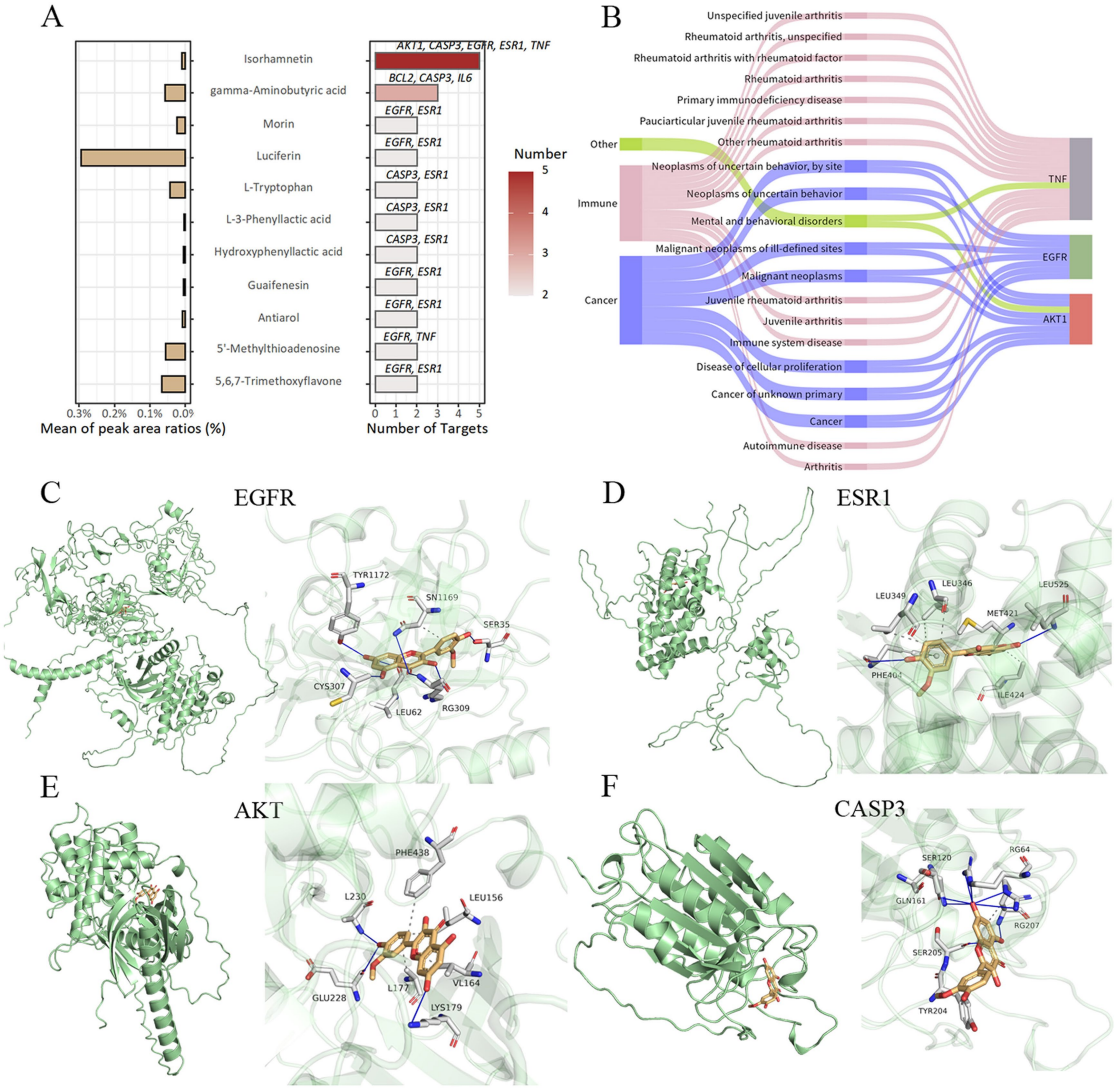
Polypharmacological landscape and molecular docking validation of isorhamnetin with core target proteins. **(A)** Dominant bioactive components ranked by the number of core targets bound; isorhamnetin binds the most hub targets among all 54 orally bioavailable compounds. **(B)** Sankey diagram illustrating specific associations between core target proteins and inflammation- or cancer-related diseases. **(C–F)** Molecular docking patterns and predicted binding conformations of isorhamnetin with four core target proteins: **(C)** EGFR, **(D)** AKT1, **(E)** ESR1, and **(F)** CASP3. Docking was performed using AutoDock Vina (v1.2.0) with AlphaFold-predicted protein structures. Predicted binding affinities (kcal/mol) are indicated for each complex.

The compound-pathway-disease landscape, where bioactive compounds converge on core targets (EGFR, AKT1) linking inflammatory and oncogenic pathways, is presented in Supplementary Figure S3. Figure 6A identifies isorhamnetin as the compound engaging the largest number of core targets (EGFR, AKT1, ESR1, CASP3) in the network model. Disease-specific analysis showed associations of autoimmune disorders with TNF/ESR1, and malignant neoplasms with EGFR/AKT1 (Figure 6B). Molecular docking revealed that isorhamnetin binds to four core targets with predicted binding affinities ranging from −6.762 to −8.341 kcal/mol (Figures 6C–F; Supplementary Table S2). These binding energies suggest moderate binding interactions and are comparable to those reported for similar flavonoid-compound interactions. However, in silico docking affinities alone do not directly confirm biological activity, and the predicted interactions require experimental validation in future studies. Docking parameters included grid boxes of 20x20x20 Angstrom centered on p2rank-predicted binding pockets with exhaustiveness set to 8. All interactions involved hydrogen bonding and hydrophobic contacts with key residues in the predicted binding pockets.

### 3.5 Experimental validation of anti-inflammatory effects in LPS-stimulated cells

Figure 1 shows the anti-inflammatory effect of isorhamnetin in LPS-stimulated Calu3 cells. Isorhamnetin treatment dose-dependently reduced the secretion of pro-inflammatory cytokines IL-6, TNF-*α*, and IL-1β compared with the LPS model group (Figures 1A–C). RT-qPCR analysis demonstrated that isorhamnetin significantly downregulated mRNA expression of pro-inflammatory genes (IL-18, IL1β, IL6, TNF-α) (Figure 1D), whereas IL-10 (anti-inflammatory cytokine) expression showed no significant changes. Western blot analysis of NF-κB signaling (Figure 1E) showed that isorhamnetin significantly and dose-dependently reduced p-p65 protein expression compared with the LPS model group (Figure 1F), indicating that the anti-inflammatory effect of isorhamnetin is mediated, at least in part, through inhibition of p65 phosphorylation.

### 3.6 Experimental validation of cancer cell apoptosis

Isorhamnetin treatment induced a concentration-dependent increase in cell mortality, which reached 37.5% at 100 μg/mL, with an IC₅₀ value of 111.8 μg/mL (Figure 2A). Fluorescence staining demonstrated that intracellular ROS levels were elevated (indicated by intensified green fluorescence) in parallel with increasing isorhamnetin concentrations (Figure 2B). RT-qPCR analysis revealed concentration-dependent upregulation of P21 and P53 mRNA expression (Figure 2C). Immunofluorescence staining showed enhanced *γ*-H2AX fluorescence intensity (Figure 2D), and RT-qPCR further confirmed upregulation of the DNA damage response genes CHEK2 and ATM, accompanied by downregulation of BRCA1 (Figure 2E), indicative of activated DNA damage signaling. JC-1 staining displayed enhanced green fluorescence in treated cells, signifying mitochondrial depolarization (Figure 2F). RT-qPCR revealed upregulation of Bax, Cycs, and Drp1, as well as downregulation of Bcl2 and Mfn2 (Figure 2G), suggesting mitochondrial dysfunction and activation of the intrinsic apoptotic pathway. Western blot analysis (Figure 2H) showed reduced phosphorylation of AKT1 (p-AKT1) at 100 μg/mL (Figure 2I), together with elevated levels of cleaved caspase-3 relative to *β*-actin and total caspase-3 (Figures 2J,K), collectively confirming modulation of AKT signaling and execution of apoptosis.

## 4 Discussion

In this study, we combined untargeted LC-MS/MS metabolomics, network pharmacology, and *in vitro* experiments to identify bioactive constituents of *L. ruthenicum* and evaluate their activities in a cell model. Our metabolomic analysis detected 149 metabolites. Organic acids, carbohydrates, and amino acid derivatives dominated in abundance, whereas flavonoids showed the highest chemical diversity (*n* = 34) but contributed only 8.17% of the total peak area. Computational screening prioritized 54 orally bioavailable compounds and 630 protein targets. Network analysis revealed ten core hub targets (AKT1, TNF, IL6, EGFR, BCL2, SRC, CASP3, ESR1, NFKB1, ALB), with AKT1 and TNF showing the highest degree centrality. Among all compounds, isorhamnetin engaged the largest number of core targets in the network model (EGFR, AKT1, ESR1, CASP3) with predicted docking affinities (−6.76 to −8.34 kcal/mol) in the moderate range. Cell-based experiments showed that isorhamnetin suppresses pro-inflammatory cytokines, induces ROS accumulation, triggers the DNA damage response, disrupts mitochondrial membrane potential, and activates caspase-3-dependent apoptosis with modulation of the AKT signaling pathway in Calu3 cells.

Anti-inflammatory mechanism. Previous studies have reported that *L. ruthenicum* extracts lower pro-inflammatory cytokines in various models (8–10). Our results are consistent with those observations: isorhamnetin dose-dependently reduced IL-6, TNF-*α*, and IL-1β secretion and downregulated their mRNA levels. However, we did not detect a significant change in IL-10 expression. Western blot analysis revealed that isorhamnetin significantly and dose-dependently suppressed p-p65 levels (Figures 1E,F), indicating that the anti-inflammatory effect of isorhamnetin is mediated, at least in part, through inhibition of NF-κB p65 phosphorylation. Alternative pathways may also contribute, such as inhibition of TLR4/MD-2 dimerization (19), modulation of AP-1 or Mincle/Syk signaling (20, 21), or activation of Nrf2/HO-1 (22, 23). Future studies using pathway-specific inhibitors would further clarify the relative contribution of each pathway.

Pure isorhamnetin showed a concentration-dependent antiproliferative effect against Calu3 cancer cells, with an IC50 of 111.8 μg/mL (approximately 370 μM). This IC50 value is modest compared to established anticancer agents; however, it is within the range reported for other dietary flavonoids in various cancer cell lines (quercetin IC50 50–200 μM in lung cancer cells). To evaluate the safety profile of isorhamnetin, we assessed its cytotoxicity on normal bone marrow mesenchymal stem cells [hMSCs; purchased from Procell Life Science & Technology Co., Ltd., Wuhan, China (Supplementary File S2)]. Isorhamnetin showed no significant cytotoxicity at concentrations up to 600 μg/mL, indicating an IC50 exceeding 600 μg/mL in normal cells (Supplementary Figure S4). This yields a selectivity index (SI > 600/111.8 ≈ 5.4), suggesting preferential cytotoxicity toward Calu3 cancer cells over normal hMSCs. This selectivity ratio is comparable to or greater than that reported for other dietary flavonoids: for example, isorhamnetin exhibited SI values of 3.0–5.3 in HepG2/LO2 cell pairs (24) and 2.5–4.8 in various cancer/normal cell models, while quercetin and luteolin have been reported with SI values of 2.0–4.5 in lung cancer models. These comparisons support the potential safety margin of isorhamnetin, although further systematic comparisons across multiple cancer and normal cell lines are warranted. As previously reported, the crude extract of *Lycium ruthenicum* Murr. inhibited tumor cell proliferation with an EC50 of 4.08 mg/mL (11). The relative content of isorhamnetin in the black goji berry extract was determined to be 0.01% (Supplementary Information S1). Based on this content ratio, the actual concentration of isorhamnetin provided by the extract at its EC50 was substantially lower than the IC50 of pure isorhamnetin. These results indicate that the antiproliferative activity of the total extract cannot be explained by isorhamnetin alone, and that synergistic effects between isorhamnetin and other active components likely contribute to the overall anticancer efficacy of *Lycium ruthenicum* Murr. Therefore, isorhamnetin may serve as a useful activity marker reflecting the antiproliferative potential of black goji berry extracts, rather than the sole active constituent. Regarding cell selectivity, several studies have shown that isorhamnetin and related flavonoids exhibit preferential cytotoxicity toward cancer cells over normal cells through mechanisms involving differential ROS metabolism and mitochondrial sensitivity (24–28). Future comparative studies using normal cell lines would further substantiate the selectivity profile.

Consistent with these molecular changes, Western blot analysis (Figure 2H) showed reduced phosphorylation of AKT1 (p-AKT1) at 100 μg/mL (Figure 2I), together with elevated levels of cleaved-caspase-3 relative to *β*-actin and total caspase-3 (Figures 2J,K). Densitometric quantification was performed using ImageJ software, with band intensities normalized to beta-actin loading control. The quantified ratios are presented in the corresponding bar graphs in Figures 2I,K, confirming modulation of AKT signaling and execution of apoptosis.

Our integrative approach was designed to prioritize compounds with favorable oral bioavailability and multi-target engagement. Isorhamnetin met these computational criteria, and its experimental effects partially overlapped with the reported activities of crude *L. ruthenicum* extracts (2, 29). We therefore consider isorhamnetin a representative bioactive flavonoid that contributes to, but does not exclusively account for, the anti-inflammatory and anticancer properties of black goji berry. Other flavonoids, phenolic compounds, or polysaccharides may act synergistically or additively. The current study focused on small-molecule metabolites; macromolecular components such as polysaccharides were not captured by our extraction and LC-MS workflow and warrant separate investigation.

Several limitations of this study should be acknowledged. First, the cell experiments used purified isorhamnetin rather than whole fruit extract; this design was intentional to validate the computational prediction, but it does not directly test potential synergistic effects among multiple constituents. Second, all experiments were conducted using a single cell line (Calu3), which limits the generalizability of the findings. The anti-inflammatory experiments should be verified in other cell types (macrophages or primary immune cells), and the anticancer findings should be confirmed in additional cancer cell lines and normal cell controls. Third, we did not perform *in vivo* pharmacokinetic or efficacy studies, which are essential for evaluating therapeutic potential. Fourth, the computational predictions—including network pharmacology and molecular docking—are inherently limited by database completeness, prediction algorithm accuracy, and the absence of experimental confirmation for most predicted compound-target interactions. Fifth, no pathway inhibition (rescue) experiments were conducted, so the cause-and-effect relationship between AKT signaling modulation and the observed cellular effects remains correlative rather than definitive. Sixth, the relatively high isorhamnetin concentrations used *in vitro* (up to 100 μg/mL) may exceed physiologically achievable levels following oral administration, and future studies should include pharmacokinetic assessment to determine relevant concentrations. Finally, although the hMSC assay (Supplementary Figure S4) suggested preferential cytotoxicity toward cancer cells (SI > 5.4), the modest IC50 value highlights the need for further optimization and mechanistic clarification. Future work could employ physiologically based pharmacokinetic modeling, tissue-specific pathway analyses, and in vivo efficacy studies to translate these preliminary findings toward functional food or nutraceutical applications.

Several directions follow from this work. The 3′,4′-dimethoxyflavone scaffold of isorhamnetin could serve as a starting point for structure–activity relationship studies. The docking models in this study may help guide initial modifications to the B-ring substituents, potentially leading to derivatives with improved target engagement profiles. On the methodological side, the integrated strategy used here, combining untargeted metabolomics with computational screening and focused *in vitro* assays, could be adapted to other medicinal or food plants where the link between composition and bioactivity is unclear. From a practical standpoint, isorhamnetin might be considered as a potential chemical marker for quality assessment of *L. ruthenicum* derived products, which could facilitate batch-to-batch consistency in future functional food development.

## 5 Future perspectives

LC-MS/MS metabolomics identified 149 metabolites in *Lycium ruthenicum* Murr., with flavonoids as the most structurally diverse class. Combined metabolomics and network pharmacology identified isorhamnetin as a bioactive component of black goji berry. *In vitro* experiments confirmed that isorhamnetin inhibits LPS-induced inflammation and suppresses Calu-3 cell proliferation by inducing ROS-mediated DNA damage, mitochondrial dysfunction, and caspase-3-dependent apoptosis via regulating the AKT signaling pathway. Moreover, the bioactivity of *Lycium ruthenicum* extract depends on the synergistic effects of multiple active components, and isorhamnetin can serve as a core flavonoid biomarker for its functional efficacy.

This work provides a phytochemical and mechanistic basis for the anti-inflammatory and anticancer potential of *Lycium ruthenicum*. However, the findings are limited to in vitro validation. Future studies will focus on *in vivo* mechanistic verification, systematic exploration of the synergistic mechanisms of multiple ingredients, and evaluation of isorhamnetin as a quality marker to further support the development and application of Lycium *ruthenicum* as a functional food resource.

## Data Availability

The original contributions presented in the study are publicly available. This data can be found here: NGDC, OMIX018521.

## Footnotes

1
http://bionet.ncpsb.org.cn/batman-tcm/

2
https://lmmd.ecust.edu.cn/netinfer/
