# Supplementary material for: Isorhamnetin from Lycium ruthenicum Murr. exhibits anti-inflammatory and anticancer activities in vitro via regulation of oxidative stress, mitochondrial function, and AKT signaling
Source: Front Nutr. 2026 Jul 23;13:1750263. doi: 10.3389/fnut.2026.1750263 (PMC13442899; doi:10.3389/fnut.2026.1750263)
Supplement: Supplementary file 1 [file Supplementary_file_1.DOCX]

Isorhamnetin from *Lycium ruthenicum* Murr. Exhibits Anti-Inflammatory and Anticancer Activities via Regulating Oxidative Stress, Mitochondrial Function, and AKT Signaling

Lei Jiang^1*^, Jideng Ma^2†^, Zhiyuan Li^3^,Yuqi Li^1^,Shihan Zhang^3^,Huifeng Li^3^

^1^Key laboratory of Tibetan medicine research, Northwest Plateau Institute of Biology, Chinese Academy of Sciences, Xining 810001, PR China;

^2^Clinical Laboratory Department, Qinghai Provincial Peoples Hospital, Xining 810007, China

^3^College of Agriculture and Animal Husbandry, Qinghai University,Xining 810001, China

***Correspondence:**

Lei Jiang

Jianglei@nwipb.cas.cn; Tel: +86 15297217193

^†^Lei Jiang and Jideng Ma contributed equally to this work

Keywords:Lycium ruthenicum Murr.; Isorhamnetin; LC‑MS/MS; Anti‑inflammatory activity; Anticancer activity; Cell apoptosis

**Figure S1. Multi-layered compound-target interaction network of *Lycium ruthenicum* phytochemicals.**

Notes：Nodes represent distinct entities: teal circles denote 54 orally bioavailable compounds (screened via RDKit, compliant with Lipinski’s rules and structural safety thresholds); orange diamonds represent 630 unique protein targets (integrated from BATMAN-TCM [236 experimentally supported targets] and NetInfer [456 high-confidence predictions with Score ≥ 1], after deduplication). Edges indicate predicted interactions between compounds and their cognate targets. Network metrics (e.g., average node degree, betweenness centrality, hub target identification) are embedded to facilitate prioritization of biologically relevant targets for functional validation.

**Figure S2. PPI network and core target analysis of *Lycium ruthenicum* related targets.**

Notes：Global PPI network， Nodes = targets; edges = high-confidence interactions (score ≥ 700). Nodes arranged by degree centrality, core targets in inner circles (high connectivity).

**Figure S3. Integrated network**

Notes：Integrated network showing interactions among bioactive compounds, core targets, signaling pathways, and associated diseases.

**Table S2. Information of RMSDs**

| Protein | File | RMSD |
| --- | --- | --- |
| AKT1 | AKT1_Isorhamnetin.pdb | 15.534 |
| CASP3 | CASP3_Isorhamnetin.pdb | 14.652 |
| EGFR1 | EGFR1_Isorhamnetin.pdb | 24.332 |
| ESR1 | ESR1_Isorhamnetin.pdb | 22.371 |
| TNF | TNF_Isorhamnetin.pdb | 15.967 |

**Table S3 .Information of primers**

| Gene Symbol | NCBI RefSeq | Forward Primer (5-3) | Reverse Primer (5-3) | Amplicon (bp) |
| --- | --- | --- | --- | --- |
| IL18 | NM_001562 | GCTGAACCCAGTCAGACTCTT | CTGTTACCAGCAAGTGTACTTCC | 131 |
| IL1B | NM_000576 | ATGATGGCTTATTACAGTGGCAA | GTCGGAGATTCGTAGCTGGAT | 132 |
| IL6 | NM_000600 | ACTCACCTCTTCAGAACGAATTG | CCATCTTTGGAAGGTTCAGGTTG | 149 |
| TNF | NM_000594 | CCTCTCTCTAATCAGCCCTCTG | GAGGACCTGGGAGTAGATGAG | 220 |
| IL10 | NM_000572 | GACTTTAAGGGTTACCTGGGTTG | TCACATGCGCCTTGATGTCTG | 112 |
| CDKN1A | NM_000389 | TGTCCGTCAGAACCCATGC | AAAGTCGAAGTTCCATCGCTC | 117 |
| TP53 | NM_000546 | CAGCACATGACGGAGGTTGT | TCATCCAAATACTCCACACGC | 133 |
| CHEK2 | NM_007194 | ATGTCGGAGGCCAAAATGTTC | CAGCGTTTCCTCCTCATACTG | 119 |
| ATM | NM_000051 | ACTGAGGTTCAAAACGCCTGT | TCCATTCATATCTGCCTAATTCCT | 84 |
| BRCA1 | NM_007294 | ACCTTGGAACTGTGAGAACTCT | TCTTGATCTCCCACACTGCAATA | 130 |
| BAX | NM_004324 | CCCGAGAGGTCTTTTTCCGAG | CCAGCCCATGATGGTTCTGAT | 155 |
| CYCS | NM_018947 | CTTTGGGCGGAAGACAGGTC | TTATTGGCGGCTGTGTAAGAG | 117 |
| DNM1L | NM_005690 | CCTGACATCATGCCGATCCT | GCTTTCTCTGTCTTGTACTTCC | 88 |
| BCL2 | NM_000633 | GGTGGGGTCATGTGTGTGG | CGGTTCAGGTACTCAGTCATCC | 89 |
| MFN2 | NM_014874 | CTCTCGATGCAACCTCTCCA | CCTGAATCAGACACTGGCTCT | 94 |
| GAPDH | NM_002046 | GGAGCGAGATCCCTCCAAAAT | GGCTGTTGTCATACTTCTCATGG | 197 |

**Figure S4. Cytotoxicity of isorhamnetin in normal human mesenchymal stem cells (hMSCs)**

Notes：(A) hMSCs were incubated with serially elevated concentrations of isorhamnetin (0 to 600 µg/mL) for 24 h, and cell viability was determined via the MTT assay. No obvious cytotoxic effect was detected at concentrations up to 600 µg/mL, suggesting that the IC50 value is higher than 600 µg/mL.

(B) Cell death was analyzed by flow cytometry using Annexin V/PI dual staining. No prominent cell death was observed at concentrations below 600 µg/mL. This demonstrates a favorable safety margin compared to Calu3 cancer cells (IC50 = 111.8 µg/mL), yielding a selectivity index (SI) > 5.4.

**Figure S5. Comparison of MS/MS fragment peaks of isorhamnetin standard and its aglycone standard.**

**Supplementary File S1. Calu3 cell authentication report. This document includes short tandem repeat (STR) profiling and mycoplasma testing results for the Calu3 (human lung adenocarcinoma) cell line used in this study, issued by Procell Life Science & Technology Co., Ltd. (Wuhan, China).**

**Supplementary File S2. hMSC (human bone marrow mesenchymal stem cell) authentication report. This document includes CD90 immunofluorescence staining results for the hMSCs used in this study, issued by Procell Life Science & Technology Co., Ltd. (Wuhan, China).**
